# Development of a helper cell-dependent form of peste des petits ruminants virus: a system for making biosafe antigen

**DOI:** 10.1186/s13567-015-0231-y

**Published:** 2015-09-23

**Authors:** Jana Baron, Michael D. Baron

**Affiliations:** The Pirbright Institute, Ash Road, Pirbright, Surrey GU24 0NF UK

## Abstract

Peste des petits ruminants (PPR) is a viral disease of sheep and goats that is spreading through many countries in the developing world. Work on the virus is often restricted to studies of attenuated vaccine strains or to work in laboratories that have high containment facilities. We have created a helper cell dependent form of PPR virus by removing the entire RNA polymerase gene and complementing it with polymerase made constitutively in a cell line. The resultant L-deleted virus grows efficiently in the L-expressing cell line but not in other cells. Virus made with this system is indistinguishable from normal virus when used in diagnostic assays, and can be grown in normal facilities without the need for high level biocontainment. The L-deleted virus will thus make a positive contribution to the control and study of this important disease.

## Introduction

Peste des petits ruminants (PPR) is a severe disease of sheep and goats which has been spreading extensively over the past two decades, and is now found widely distributed through large parts of Africa, the Middle East and Asia; it poses an increasing threat to poor livestock keepers, primarily in developing countries [1–4]. The disease is caused by a virus, PPR virus (PPRV), which is a morbillivirus, related to the human pathogen measles virus (MV), as well as other animal pathogens such as canine distemper virus (CDV) and the now-eradicated rinderpest virus (RPV). Control of this disease has recently become a major international goal, marked by the adoption in 2014 of a resolution by the World Organisation for Animal Health (OIE) to establish a control programme with a view to eventual eradication of the disease [5].

Disease control is mostly achieved through the use of clinical or laboratory-based diagnosis coupled with vaccination. All the vaccines currently in use are attenuated strains of PPRV [6,7]; these vaccines are effective, though they do not provide a DIVA (Distinguishing Infected from Vaccinated Animals) capability, since they provide what is essentially a totally subclinical infection with PPRV, and the antibody signatures of vaccinated and previously-infected animals are identical. Several alternative DIVA vaccines have been proposed based on recombinant viruses [8–13], but none is yet in field use.

The most commonly used laboratory tests are those for anti-PPRV antibodies, partly on cost grounds, and partly because a lot of the effort in infected countries is still on tracking the prevalence of disease through identifying flocks/herd which have been exposed to the virus, rather than acute response diagnostics on animals showing clinical signs. In addition, vaccination programs are increasingly being supported by post-vaccination serum surveillance to measure the effectiveness of the local vaccination process. Although there are still laboratories using agarose gel immunodiffusion (AGID) techniques, the primary method of testing for anti-PPRV antibodies is competition ELISA (cELISA), and the antibodies tested for are either those recognising the viral nucleocapsid protein (N) [14,15], in which the ELISA antigen is a bacterially expressed protein, or those recognising the viral surface glycoprotein H [16,17], where whole virus is used as the ELISA antigen. The latter system presents a problem in the need to grow and purify live virus, even if it is only a vaccine strain of the virus. In many countries, this requires a containment laboratory for the virus culture, and continued application of biosecurity restrictions, including restrictions on the transport of the ELISA kits, even if the virus preparation is subsequently inactivated, e.g. with binary ethylenimine (BEI). For larger scale and simplified production of antigen for ELISA, it would be useful to be able to prepare a suitable antigen without the need for actual virus. The H protein requires mammalian glycosylation for proper folding, so baculovirus-expressed protein is not adequate. We have therefore sought to create a biosafe system to produce virus-like particles (VLPs) which would appear as virus in diagnostic tests and which could be produced in good yield. We have achieved this by deleting an essential gene from the PPRV genome and providing the required protein *in trans* in a helper cell line.

In our previous studies, it was shown that removing the P gene from a morbillivirus genome and providing the P protein from a helper cell line can lead to the production of suitable VLPs, but in too low yield for practical use, since the P-expressing cell line cannot synthesize the amount of P protein that is required to support normal virus replication and assembly [18]; the P gene is, in any event, a complex system in morbilliviruses, being also required for the production of accessory proteins V and C, both of which are required for optimal virus replication [18–21]. We show here that a PPRV genome lacking the entire gene for the viral polymerase (L) protein grows to near-normal levels in a cell line expressing the L protein, generating VLPs that act as a good antigen in the cELISA, as well as acting as a model virus for detection by PCR-based techniques. The VLPs cannot replicate in other cell lines.

## Materials and methods

### Cells and viruses

Vero cells expressing the canine version of the morbillivirus receptor SLAM (Signalling Lymphocyte Activation Molecule) (Vero-Dog-SLAM, VDS) were obtained from Dr Paul Duprex, then at Queen’s University Belfast, N. Ireland, and maintained in Dulbecco’s modified Eagle’s medium containing 25 mM HEPES buffer, penicillin (100 U/mL), and streptomycin (100 μg/mL) (DMEM) containing 10% foetal calf serum (FCS). Zeocin was included at 0.1 mg/mL to maintain selection for SLAM expression. PPRV Nigeria 75/1 vaccine strain [6] and recombinant PPRV rPPRV-GFP [22] were propagated and titrated in VDS cells. Titres were determined as the 50% tissue culture infectious dose (TCID_50_), calculated by the method of Spearman and Kärber [23]. Titres of preparations of PPRV-del-L were determined in VDS-L cells, while titres of complete PPRV were determined in VDS cells.

### Transfections and infections of cultured cells

VDS cells or VDS-L cells were plated at 4 × 10^4^ cells per well in 12-well plates or 10^5^ cells per well in 6-well plates. For immunofluorescence studies, the wells contained sterile glass coverslips (10mm in diameter). The cells were allowed to attach and were infected 6-18 h after plating at a multiplicity of infection (moi) as indicated for each experiment. For infection studies, virus inocula were removed after one hour, the cells washed once with phosphate buffered saline and fresh medium added. Cells were transfected using TransIT-LT1 according to the manufacturer’s instructions, using 3 μL transfection reagent per μg DNA. At the indicated time after transfection, cells were fixed for immunofluorescence staining and confocal microscopy [22], or dissolved in SDS-PAGE sample buffer, or subjected to a freeze-thaw cycle to release progeny virus.

### Recombinant PPRV del-L

The PPRV genomic clone (pPPRV-GFP) and the procedure for recovering live virus from plasmids was as previously published [22]. The PPRV-del-L genome was constructed by removing bases 10031-16690 from the PPRV-GFP cDNA, i.e. the entire sequence from 26 bases after the H open reading frame (ORF) to 5 bases after the L ORF. The sequences removed include the H gene stop signal, the intergenic trinucleotide, the L gene start signal and the whole L ORF (a total of 6660 bases). This genome manipulation was carried out using Gibson assembly [24] of two large PCR products derived from the PPRV-GFP plasmid. The two fragments were generated using primer pairs Del-L_F1 [TGCAACCATCGCTCGAGCAAGTGATACATCTGCCCCCTTCTC] with Del-L_R1 [GATTCTTGTGTCAACCCCTGGA] and Del-L_F2 [TCCAGGGGTTGACACAAGAATC] with Del-L_R2 [GAGAAGGGGGCAGATGTATCACTTGCTCGAGCGATGGTTGCA]. The entire sequence of the resulting PPRV-del-L genome was checked.

### Creation of the helper cell line

To create the cell line expressing PPRV L, the exact L ORF, without any transcription control sequences (i.e. no gene start, gene stop or untranslated region), was fused downstream of the V5 epitope tag and cloned under the control of the promoter in pCAGGS (pCAGSS-V5-L). A large section of this plasmid, consisting of the pCAGGS promoter and intron and the V5-L ORF, was then inserted into plasmid 5’-PTK-3’ [25], upstream of the IRES-driven puromycin resistance marker in that vector. To do this, the En-2 splice acceptor was removed from 5’-PTK-3’ and replaced with a short multiple cloning site (*XhoI/SgfI/AflII/SbfI/NheI/MfeI*) to give pPB-MCS-IPUR. The CAGGS-V5-L construct from pCAGGS-V5-L was then inserted into pPB-MCS-IPUR to give pPB-V5-PPRV-L. The plasmid pPB-V5-PPRV-L was transfected into VDS by standard techniques along with plasmid pCyL43 encoding the transposase [26] and the cells subjected to puromycin selection through 3 passages to create the VDS-L cell line.

### Other assays

RNA was extracted from cells using the Qiagen RNeasy mini kit. cDNA was synthesised using Superscript II reverse transcriptase with oligo(dT)-anch [27] as the primer. Real-time quantitative PCR of PPRV N mRNA or host cell L13A mRNA was carried out essentially as previously described [18,27], using a set of primers recognising the PPRV N gene and optimised for SYBR Green-based qPCR (NF2b:CGGGTTGACCTTTGCATCA and NREVb: CTTTGTTGTGTGTATTTAACCCACCTT). Confocal microscopy was performed as previously described [22]. Mouse anti-V5 tag was obtained from AbD Serotec and AlexaFluor 568 anti-mouse IgG was from Life Technologies. All images were taken by sequential scanning and the resulting separate colour images overlayed in Photoshop. cELISA antigen was prepared as previously described [16]. SDS-PAGE and Western blots were carried out as previously described [28], except that the Western blot transfer was performed using a TransBlot SD Semi Dry Electrophoretic Transfer Cell (Bio-Rad) and Bjerrum and Schafer-Nielsen transfer buffer (48 mM Tris, 39 mM glycine, 37.5 mg/L SDS, 20% methanol, pH 9.2) [29]. Statistical analysis was performed in Minitab v17.

## Results

**Figure 1:**
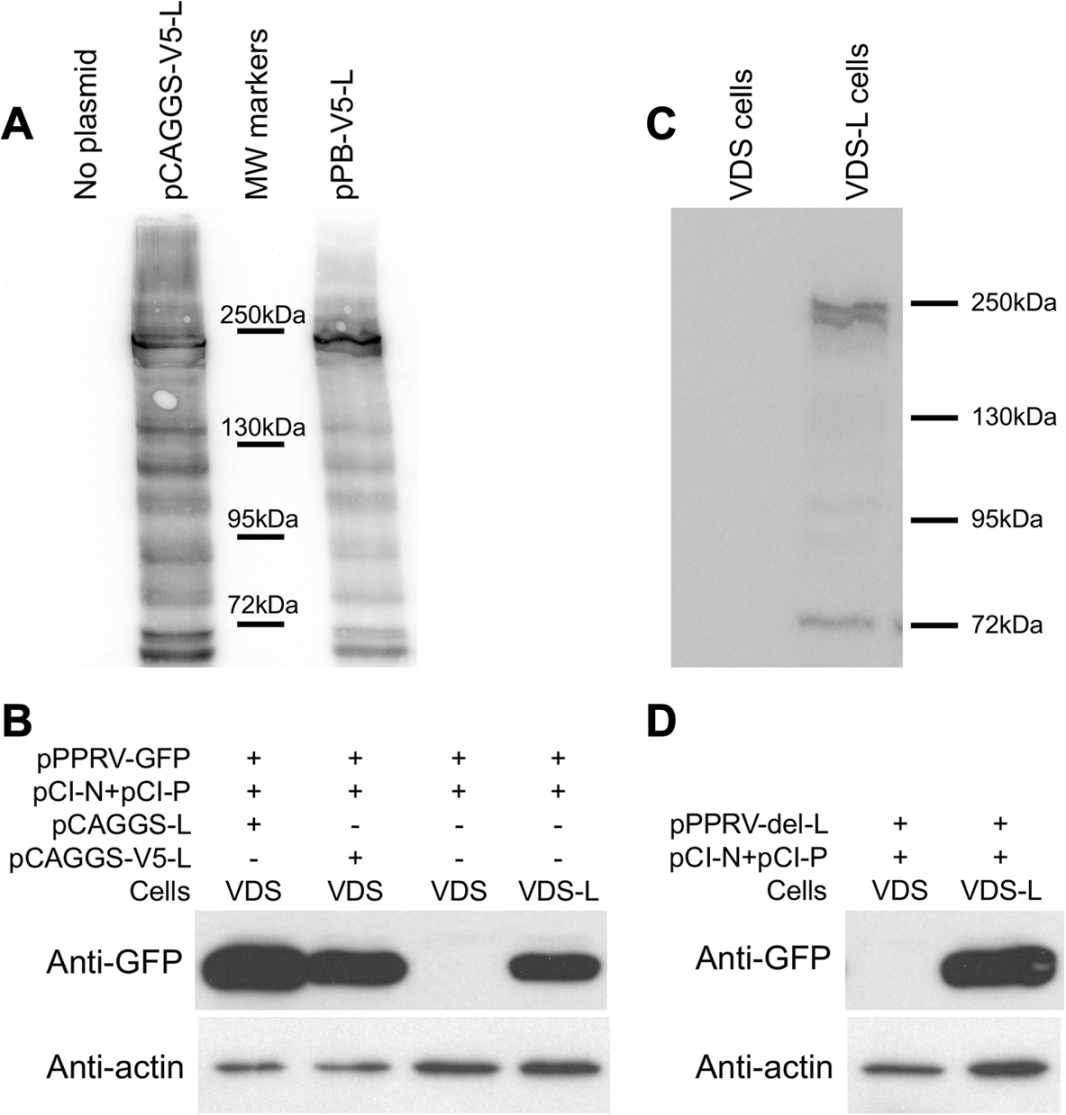
**Expression of functionally active PPRV L protein. A** VDS cells were transfected with the indicated plasmid (2 μg) in 6-well dishes and cultured for 48 h. After lysis of the transfected cells, expressed proteins were analysed by SDS-PAGE and Western blot using mouse anti-V5 tag. **B** Cells (VDS or VDS-L) in 6-well dishes were transfected with the indicated plasmids and incubated for 8 days. The transfected cells were subjected to 1 freeze-thaw cycle to release infectious virus and the clarified supernatant used to infect fresh VDS or VDS-L cells. After 7 days, the cells were harvested and the expression of virally-encoded GFP analysed by SDS-PAGE and Western blot. Actin was used as a loading control. **C** VDS or VDS-L cells (~4 × 10^5^) were lysed directly in SDS-PAGE sample buffer and analysed by SDS-PAGE and Western blot using mouse anti-V5 tag. **D** As B, except that no plasmid encoding the L protein was included.

In previous work, it was found that a recombinant rinderpest virus in which the L protein had an amino-terminal HA tag was fully functional (Baron, M.D., unpublished). It seemed likely that insertion of an epitope tag at the amino-terminus of a morbillivirus L protein does not interfere with its function, so a PPRV L protein expression construct was created with the V5 epitope tag at this position. Despite the large size of the L protein, significant amounts of full-length L protein were detected in cells transfected with this construct (Figure 1A). In addition, the V5-L construct was equally effective in supporting the rescue of PPRV from the plasmid copy of the genome as was the original pCAGGS-L (Figure 1B), confirming that it is functional as the viral polymerase.

Since the V5-L was fully functional, the VDS-L helper cell line was created using this construct. VDS cells were selected as the base for the new cell line as they have been, in our hands, the best cell line for growing PPRV, either wild-type or vaccine strains. The presence of the morbillivirus receptor (SLAM) ensures that virus growth leads to rapid production of large syncytia, which are easy to detect and characteristic of virus replication and viral glycoprotein expression. Normal methods for creating cell lines expressing extra proteins by transfecting with the plasmid of interest, in linear or circular form, along with a plasmid encoding an antibiotic resistance marker, were ineffective. Resistant cells could be isolated, but they either did not express any V5 tag-bearing protein at all or the protein detected was a severely truncated form of the L protein. Since a problem with these methods is that they rely on random recombination events of genomic DNA with the plasmids in question, it appeared that the L sequence was long enough to make it highly probable that such an event would occur within its sequence, or that of the governing promoter. We therefore sought to carry out a directed insertion of a defined expression cassette using the PiggyBac transposase system [30]. This method was very effective. The resultant cell line was isolated at the first attempt and was found to express an L protein that was the same size as the original (Figure 1C), and was able to support rescue of rPPRV-GFP when the cell line was the only source of L protein (Figure 1B), showing that the cell line-expressed L protein was functional.

**Figure 2:**
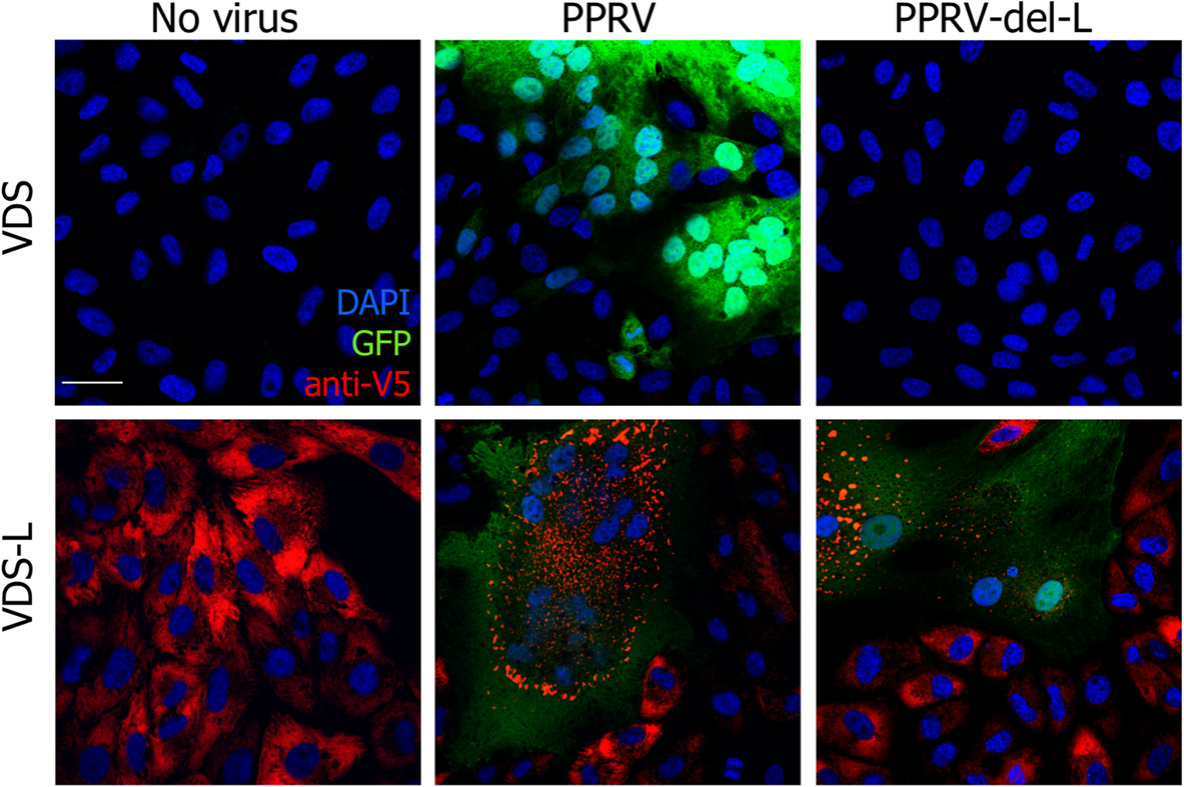
**PPRV-del-L requires helper cell line.** VDS or VDS-L cells were infected with PPRV-GFP or PPRV-del-L at moi = 0.01 and incubated for 48 h. Cells were fixed and stained with mouse anti-V5 tag and AlexaFluor 568-conjugated goat anti-mouse IgG. Nuclei were stained with DAPI. Confocal images were taken by sequential scanning.

A corresponding version of pPPRV-GFP was then created which lacked the L gene. In order to preclude any possibility of the full virus being recreated by recombination with L sequences in the cell line, we deleted not only the L ORF, but also all the transcriptional control sequences required for its transcription by the viral RNA-dependent RNA polymerase. Correspondingly, the construct inserted into the cell line genome contains only the L protein ORF, so there is no sequence common to the L protein mRNA and the cut-down PPRV genome. The PPRV-del-L genome could be rescued (as evidenced by expression of GFP and the viral glycoproteins and development of cytopathic effect (cpe)) in VDS-L cells, but not in the normal VDS cells (Figure 1D). The resultant virions could be harvested, titrated and passaged as for the parental virus, but only on VDS-L cells; no replication was seen in VDS cells (Figure 2).

**Figure 3:**
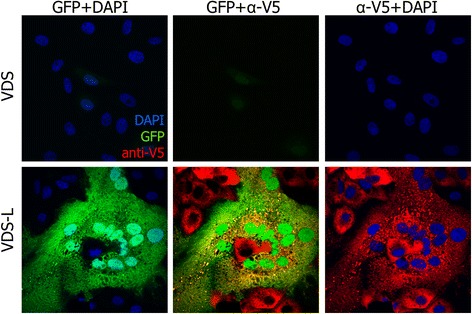
**Protein expression from PPRV-del-L after infection at higher moi.** Cells were infected with PPRV-del-L at moi = 0.5. At 48 hours post infection, cells were fixed and stained as in Figure 2. Confocal images were taken by sequential scanning. Fields showing infection and protein expression in VDS and VDS-L are shown.

Because it is a normal constituent of the virion, a small amount of the L protein was found in the PPRV-del-L VLPs produced in VDS-L cells. L protein carry-over was sufficient that a low level of GFP expression could be detected in VDS cells infected with PPRV-del-L, though only if the infection was carried out at a relatively high multiplicity of infection (moi) (Figure 3). In order to be sure that no PPRV-del-L was growing in cells without the L protein, serial passage of the VLPs on VDS cells was carried out in order to try to amplify any functional virion. No cpe was seen during these serial passages, nor was there any sign of GFP expression. Quantitative RT-qPCR was used to assay viral mRNA from each one of the passages, and showed only the expected decay of the viral RNA present in the initial inoculum.

**Figure 4:**
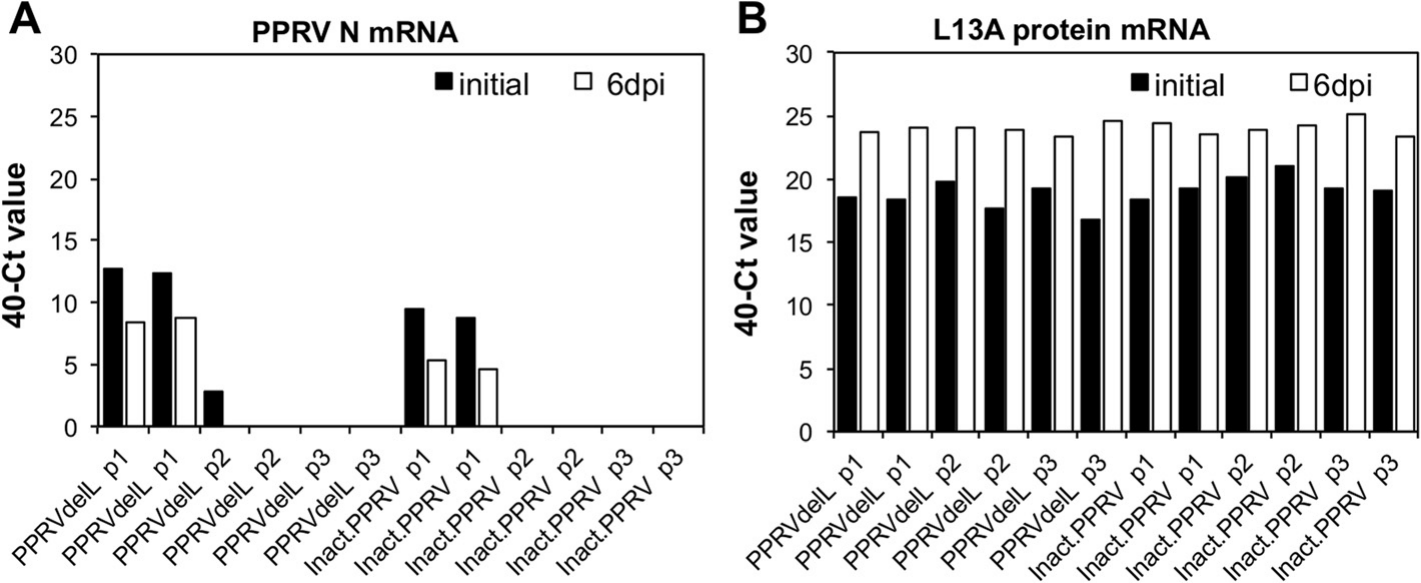
**PPRV-del-L behaves as heat-inactivated PPRV.** VDS cells were infected with PPRV-del-L (PPRVdelL) or PPRV that had been heat-inactivated (2 h at 58 °C) (inactPPRV) at a nominal moi of 0.5. After removing unattached virus, duplicate wells were harvested immediately for RNA or cultured for 6 days (p1). After 6 days, the infected cells were subjected to 1 freeze-thaw cycle, the cell pellet harvested for RNA, and one third of the supernatant passaged to fresh cells (p2). This procedure was then repeated (p3). RT-qPCR was used to determine the relative amount of viral mRNA in each sample (**A**); Vero cell L13A mRNA was used as control for the recovery of RNA from each sample (**B**). Results are expressed as 40-(mean Ct value observed from duplicate PCRs from each of duplicate wells).

No evidence for replication of the viral genome was seen in VDS cells, even with repeated passage (Figure 4). Note that for PPRV, as for other morbilliviruses, the virus inoculum is not pure virus, and the original inoculum always contains significant non-infectious viral genome and even some viral mRNA; this means that RNA extracted from virus stocks, or RNA extracted from infected cells harvested at t = 0, always gives a positive signal in RT-qPCR (Figure 4).

**Figure 5:**
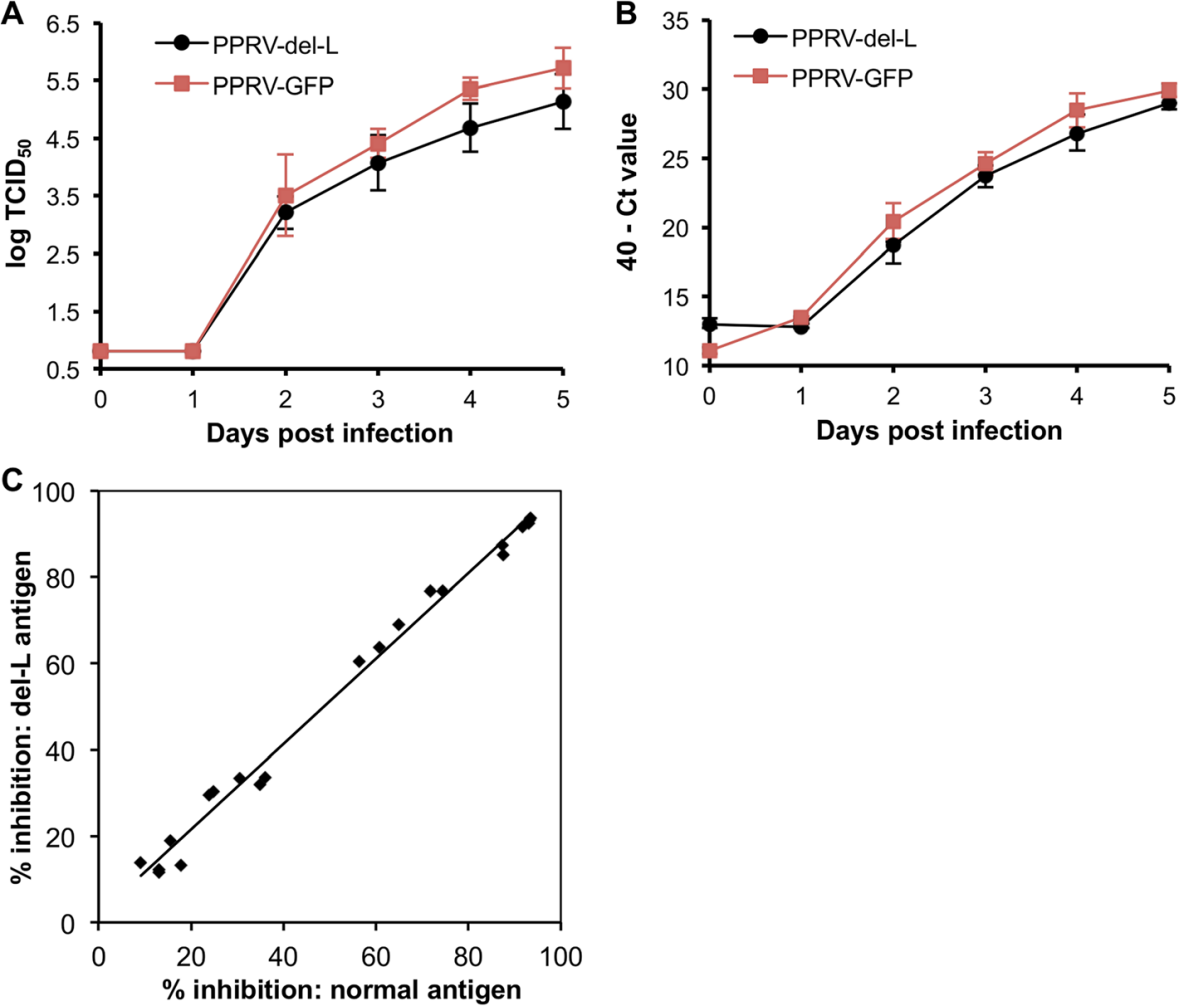
**Comparison of growth and antigenicity of PPRV-del-L and full PPRV.** VDS-L cells in 6-well plates were infected with PPRV-GFP or PPRV-del-L at moi = 0.01. Duplicate wells were harvested at 0, 1, 2, 3, 4 and 5 days post infection, subjected to one freeze-thaw cycle and the released virus titrated (**A**). RNA was extracted from the cell pellets and viral (genome + mRNA) assayed by RT-qPCR (**B**). **C** VDS-L cells were infected with PPRV-del-L (moi = 0.01) in 175cm^2^ flasks and cultured for 5 days. Virus antigen was prepared and used in the PPRV-H cELISA (BDSL) according to the manufacturer’s instructions. A set of samples known to be positive (% inhibition >50%) and negative (% inhibition <50%) were assayed in parallel cELISA tests using either the PPRV antigen provided with the kit or the antigen made from PPRV-del-L. All sera were assayed in duplicate in each test and the mean % inhibition obtained with each antigen plotted in the graph. The set of individual mean values for each serum were compared using a paired t-test: the mean difference was not significantly different from zero (*p* = 0.081). The regression line had a slope of 0.99 ± 0.02 (± S.E.) and an intercept of 1.81 ± 1.34.

Cells infected with live rPPRV-GFP show a steadily increasing amount of viral genome with time, reflecting viral replication (Figure 5). However, if the initial inoculum is heat-inactivated PPRV, the content of viral genome simply decays over time, with no indication of replication (Figure 4). The pattern seen with PPRV-del-L in VDS cells was similar to that seen with inactivated PPRV.

Multi-step growth curves were performed to compare the growth kinetics of the parental and del-L viruses. These studies were carried out in VDS-L cells, so that the same cell line was used for the two viruses. It was found that the PPRV del-L virus grew similarly to normal virus in these cells (Figure 5), although still slightly slower than the normal virus (2-way ANOVA, *p* < 0.05 for growth measured as either virus (Figure 5A) or viral RNA (Figure 5B)). Interestingly, the normal PPRV grew slightly worse in the cells expressing endogenous L protein than in parental VDS cells (compare Figure 5A with Figure 3A of [22] and Figure 2A of [18]). This observation is in accord with the observation that virus-encoded GFP expression from full PPRV is lower in VDS-L cells than in VDS cells (Figure 2). This apparent defect in virus replication in the presence of excess L protein may reflect previous observations that the correct ratio of the P and L proteins is critical in replication/transcription of paramyxovirus genomes [31,32].

Antigen for the PPRV cELISA was prepared from PPRV-del-L grown on VDS-L cells and its performance in the assay compared with that of the antigen distributed with H protein-specific cELISA kit; the results were essentially identical (Figure 5C), showing that the VLPs produce an antigen which behaves exactly like normal antigen in cELISA, while the VLPs can act as a template for PCR tests.

## Discussion

In our previous work [18], we showed that deletion of the morbillivirus P protein coding sequence produced a replicon that could replicate only in cell lines expressing the P protein, although even then with greatly reduced replication relative to the intact virus. The viruses lacking the P protein coding sequence were completely dependent on the helper cell line for transcription and replication. In the work presented here, we have shown that a replicon lacking the L (polymerase) gene (PPRV-del-L) can replicate in a cell line expressing the L protein (VDS-L), and with efficiency close to that of the parental virus. This replicon is unable to replicate in any other cell, since it lacks the L gene in its entirety. Repeated blind passage of the VLPs produced by PPRV-del-L in cultured cells showed no replication of the replicon genome (as assessed by RT-qPCR).

A key question in considering the biosafety of the PPRV-del-L is whether there is any possibility of the virus recovering its ability to replicate through the recovery of its polymerase gene or the gene for a polymerase that would fulfil the same function. Replication and transcription of PPRV, as with all nonsegmented negative-strand RNA viruses (NNSRVs), take place exclusively in the cytoplasm of the infected cell. Because the NNSRVs all co-transcriptionally encapsidate their genome and antigenome RNAs, naked viral RNAs (other than mRNAs) are not found in the cytoplasm. In addition, the template for the viral RNA polymerase is encapsidated viral RNA containing specific promoter sequences (the 5’ and 3’ terminal 120 bases), and never naked RNAs, whether viral mRNAs or derived from the host genome. For this reason, recombination is not found in this group of viruses in nature (reviewed in [33]). There is one published example of the creation of a chimeric NNSRV, which was done in culture and under specific selection for recombinant viruses [34]; in that particular case it was a combination of two different strains of respiratory syncytial virus. There is also some indirect evidence from analysis of sequences in the database that recombination between strains of the same virus has occurred in nature [35–41]; there is no evidence either in nature or in culture of recombination between dissimilar NNSRVs or between NNSRV genomes and cellular RNAs.

Given these findings, it is clearly possible for PPRV-del-L to recover its L gene through recombination with a natural PPRV virus. In this case, of course, there is already live PPRV in the system, so this circumstance would not introduce an additional hazard. If the donor is itself a wild type virus, there is already pathogenic virus present. The del-L virus is based on the fully attenuated vaccine strain of PPRV. The stability of this vaccine through several decades of use in the field shows that it has attenuating mutations throughout the genome, as was found for the Plowright vaccine [42,43], and indeed the P, F, H and N proteins of the vaccine strain all show extensive differences from those of wild type viruses isolated at the same time and geographical area, while the promoters in the vaccine show changes to bases that are conserved in all known wild type viruses. Donation of L from a different PPRV vaccine will not therefore result in a chimera that has become virulent. Recombination between the del-L virus and a different PPRV virus would in any event require coinfection of the same cells; since PPRV is restricted to containment laboratories in countries where the virus is not endemic, use of the del-L system in non-containment laboratories does not pose a threat.

Another possibility is that PPRV del-L could gain an L gene from another (non-contained) morbillivirus. This would require recombination between the PPRV genome and that of another morbillivirus (e.g. CDV, measles virus). There is no evidence from nature of recombination having occurred between two different NNSRVs which has given rise to a viable chimeric virus. This may be because such recombination has to occur at very similar sequences, as is known to be the case in pox virus recombination [44]. Alternatively, it may be because co-evolution of the viral proteins has led to incompatibilities between proteins from different, even if closely related, viruses, for example the finding that the surface glycoproteins (F and H) of PPRV will work together, but not in any heterologous pair with the H and F proteins of RPV [45]. More importantly, the N, P and L proteins of morbilliviruses, which together form the transcription/replication complexes, only work effectively when all three come from the same virus [46]. A PPRV that had acquired the L gene of CDV from replicating CDV would therefore have a non-functional N/P/L combination. Whatever the underlying biochemistry, the evidence over hundreds of years of cocirculation of different morbilliviruses (and other paramyxoviruses) is that recombination between genomes of different viruses does not occur, and the PPRV-del-L will not acquire a functional polymerase through recombination with a different morbillivirus.

A third risk might be that the del-L virus could reacquire the L gene from the helper cell line. As mentioned previously, there is no recorded case of a NNSRV picking up genetic material from a host mRNA, making it unlikely that it would happen in this case. In any event, the entire L gene transcription unit is removed from the del-L construct (including the gene transcription start and end sequences), while the helper cell contains only the L protein ORF, so that even if recombination were to occur between viral genome and cell mRNA, vital parts of the virus would be missing.

While we have not carried out this study, it is likely that the PPRV-del-L construct could be replicated by another morbillivirus, though not very well. A related construct based on two defective measles virus genomes has been propagated [47], though the resultant virus did not grow well, presumably because the minimum infectious unit needed to contain one of each genome. It was only maintained because both genomes were defective and had to support each other in trans. Co-infection of a cell by both PPRV-del-L and another morbillivirus (e.g. CDV) would lead to replication of the PPRV-del-L genome and transcription of its genes. However, this is essentially the same as the CDV replicating in the presence of a large deletion DI (defective interfering particle). Replication of the CDV would be decreased, and the “DI” still could not replicate independently. PPRV as such would not be recreated. Such a mixed infection could conceivably give rise to a sort of two-segmented pathogenic virus containing both the full genome of the non-contained morbillivirus and that of the PPRV-del-L replicon, but such a mixture would be under the usual selective pressure to lose the "DI" component.

Our results in morbilliviruses are completely in line with work done with another NNSRV, vesicular stomatitis virus (VSV), which has been developed in several laboratories as a gene delivery tool by removing the gene for the viral G protein; the VSV G protein functions both for viral attachment and fusion with the target cell. The resultant cut down viral genome, with or without the addition of other, heterologous, coding sequences (e.g. influenza HA, luciferase) could be replicated by providing the G protein in a helper cell line, which is used for growing stocks of resultant VLPs, e.g. [48–58]. This type of VSV construct is generally classified as BSL1 e.g. [49], as transmissibility is entirely dependent on the helper cell line’s provision of the G protein. A similar series of constructs has been developed based on Sendai virus (SeV), where either the F or M genes, or both, are deleted e.g. [59–66]. These constructs have been used for various functional studies as well as vaccine vector studies, and are generally recognised as nontransmissible (though risk assessment depends on the nature of the additional gene inserted into the constructs, if any).

In summary, we have created a version of a highly restricted virus which can only replicate in a defined helper cell line. The resultant construct is therefore safe to use and manufacture out of containment. It can be a safe source not only of antigen for diagnostic kits, but for validation of diagnostic assays (PCR, icELISA, VNTs) in laboratories which have restrictions on admitting live PPRV (even if subsequently chemically inactivated), or PPRV from other countries (e.g. international ring trials). In addition, the system described could be used by laboratories wishing to study replication and assembly of PPRV but lacking appropriate high containment facilities. The system has been approved by the UK Health and Safety Executive for use at BSL 1.
